# Rabies Vaccine Baits, Pennsylvania

**DOI:** 10.3201/eid1112.050685

**Published:** 2005-12

**Authors:** Virginia M. Dato, Charles Rupprecht

**Affiliations:** * Pennsylvania Department of Health, Pittsburgh, Pennsylvania, USA; † Centers for Disease Control and Prevention, Atlanta, Georgia, USA

**Keywords:** Vaccinia, rabies, vaccine, exposure, poxvirus, lyssavirus, wildlife, public health, letter

**To the Editor:** Oral rabies vaccine (ORV) programs control rabies in terrestrial reservoir species by distributing vaccine in baits (*1*). The current US-licensed ORV consists of a rabies virus glycoprotein gene inserted into the thymidine kinase gene of an attenuated strain of the Copenhagen vaccinia virus (V-RG) (*2*). Safety experience includes extensive animal studies (*2**,**3*) in which significant adverse effects were seen only with parenteral (but not mucosal) exposure of nude mice to V-RG (*4*). Usage monitoring (*4**,**5*) found only 1 human adverse complication to date (*6*).

We report our experience monitoring pet and human exposure to V-RG as part of a multiagency federal-state cooperative program that distributed 1,710,399 V-RG-laden baits from August 11, 2003, to September 17, 2003, over 25,189 km^2^ of western Pennsylvania (human population ≈3 million). The baits consisted of a vaccine-filled plastic sachet surrounded by a fishmeal polymer. Workers distributed these baits on the ground from vehicles or by air from fixed-wing aircraft using conveyor belts. Aircraft did not release baits when over homes or other areas where humans or pets were likely to be present. Given the limitations of dispersing 1,421,517 baits at a frequency of 75 to 150 baits/km^2^ from 200 m in the air, human habitat could not be totally avoided.

Each bait was printed with a toll-free phone number. Phone calls were routed to a local or district health department where an ORV-specific form adapted from the Ohio State Health Department was used to collect uniform information about bait contact.

During the 2003 campaign, Pennsylvania health departments and districts received 105 reports from persons who found 190 baits. This rate of reporting, 6.1 per 100,000 baits, is in the midrange of other published reports (0.12–50 per 100,000 baits) (*5**,**6*).

Of the 105 reports, 69 involved persons who picked up or had other skin contact with baits, and 8 reported likely contact with vaccine. Four involved persons who were hit by baits from the air. Seventy reports involved a pet or pets. In 66 reports, the pet was a dog. In 56 reports, a dog picked up the bait in its mouth. Eight of these dogs ate the bait, and another 6 ruptured the plastic sachet.

The only definite human exposure to vaccine occurred when a dog ruptured a bait and contaminated its owner's hands. Seven reports of possible human contact with vaccine involved 10 persons. No documented adverse reactions were associated with any definite or potential human exposures.

Of the 7 reports of possible human vaccine exposures, 3 incidents (4 persons) involved owners who put hands or fingers in a dog's mouth to retrieve a bait, 1 incident involved a dog that licked 2 children right after rupturing the bait, and 2 incidents (3 persons of whom 2 were children) involved picking up a potentially ruptured bait.

The final possible exposure to vaccine involved 1 of 4 persons hit by a bait. This person reported that after being struck, pink liquid spilled out of the bait. The bait was examined by program personnel and appeared to be intact. The other 3 persons (including 1 child) hit by baits did not report vaccine contact or injury.

One uninsured person, who was sent to a hospital emergency room because of potential vaccine exposure to the eye, signed out against medical advice to avoid receiving a bill. This person was seen by a family nurse practitioner 2 weeks later. Results of an examination were normal, and the person refused to have blood drawn for rabies or vaccinia titers.

Eleven children were involved in 9 credible incidents. In addition to the previously described children, 6 children picked up intact baits. We received 2 noncredible reports: a child with a dog ate a bait and a child licked a bait. In the first case, the child was in a different city at the time the alleged incident occurred, and in the second case, the caller refused to supply any information that could be used to validate the episode.

Posters, brochures, a press conference, and press releases have been used to educate the public to take precautions (for example, wash exposed skin and never remove bait from an animal's mouth) necessary to protect the most vulnerable. Callers were asked questions to determine their ORV awareness. Seventy-nine callers (75%) did not know about ORV activities, and 75 (71%) did not know what the bait was before speaking with us. Those who did know about the program had most often learned about ORV programs through paid radio announcements in neighboring Ohio.

Modifications for 2004 included an increase in media outreach in smaller markets and increased hand baiting. We received fewer reports (51, or 2.9 per 100,000 baits) of persons finding baits in 2004.
